# Higher visual gain contributions to bilateral motor synergies and force control

**DOI:** 10.1038/s41598-022-23274-x

**Published:** 2022-10-31

**Authors:** Tae Lee Lee, Hanall Lee, Nyeonju Kang, James H. Cauraugh

**Affiliations:** 1 Department of Human Movement Science, Incheon National University, Incheon, South Korea; 2 Division of Sport Science, Sport Science Institute, and Health Promotion Center, Incheon National University, Incheon, South Korea; 3 Neuromechanical Rehabilitation Research Laboratory, Division of Sport Science and Sport Science Institute, Incheon National University, 119 Academy-ro, Yeonsu-gu, Incheon, South Korea; 4 Department of Applied Physiology and Kinesiology, University of Florida, Gainesville, USA

**Keywords:** Neuroscience, Psychology

## Abstract

This study investigated the effects of altered visual gain levels on bilateral motor synergies determined by the uncontrolled manifold (UCM) hypothesis and force control. Twelve healthy participants performed bimanual index finger abduction force control tasks at 20% of their maximal voluntary contraction across four different visual gain conditions: 8, 80, 256, and 512 pixels/N. Quantifying force accuracy and variability within a trial provided a bimanual force control outcome. The UCM analysis measured bilateral motor synergies, a proportion of good variance to bad variance across multiple trials. Correlation analyses determined whether changes in the UCM variables were related to changes in force control variables from the lowest to highest visual gain conditions, respectively. Multiple analyses indicated that the three highest visual gain conditions in comparison to the lowest visual gain increased values of bilateral motor synergies and target force accuracy. The correlation findings showed that a reduction of bad variance from the lowest to three highest visual gain conditions was related to increased force accuracy. These findings reveal that visual gain greater than 8 pixels/N facilitates bimanual force control.

## Introduction

Bimanual force coordination requiring either similar or dissimilar forces between two hands is important to successfully perform many activities of daily living^1–3^. Moreover, visual feedback predominantly influences interlimb coordination patterns contributing to task performance because sensory and perception processes are closely linked with precise motor control^4,5^. For example, in the absence of visual feedback while performing isometric force control tasks, bimanual hand movements tended to show more in-phase coupling patterns that increased task error and variability. However, the presence of visual feedback altered interlimb coordination in that more anti-phase coupling patterns appeared and force control improved^6–9^. Consistent with the effects of visual feedback availability, an increased amount of visual information typically facilitates improvement in interlimb coordination patterns and force control.

Specifically, the amount of visual feedback provided during bimanual isometric force control tasks can be manipulated by changing the levels of visual gain (e.g., pixels/N)^9,10^ and/or visual angle^11,12^. The higher values of visual gain and visual angle showed that performers process the expanded visual information displaying simultaneous total forces produced by two hands relative to the targeted force level. Previous studies reported that a greater amount of visual feedback manipulated by either visual gain or visual angle effectively reduced unilateral force control error and variability^13–15^. Moreover, an increased amount of visual feedback (i.e., higher visual gain) improved bimanual force control including a reduction of force error and variability^9,16^. Presumably, the positive effects of increased visual feedback on both unimanual and bimanual force control were associated with an increased activation of the visuomotor networks^13^. Furthermore, the improved bimanual force control was accompanied with distinct interlimb force coordination patterns related to visuomotor processing. This interlimb force coordination evidence comes from the negative correlation patterns indicating error-compensatory behaviors and greater peak coherence within a delta frequency band 0–4 Hz^17–19^. These findings revealed that higher visual gain improved bimanual force control within a single trial.

According to a traditional view of human movements, execution errors can be classified into two different perspectives: (a) motor error within a trial and (b) motor errors across multiple trials^1,4,20–22^. Motor errors within a trial appear because of impaired online-motor corrections. Thus, the within-trial approach measures how the motor system simultaneously controls movement variability over time. During bimanual force control, traditional force control variables such as accuracy (e.g., root-mean squared error: RMSE) and variability (e.g., standard deviation: SD) of total force produced by two hands within a trial typically are used to characterize motor execution errors. On the contrary, motor errors across trials are observed when an inappropriate motor action is initiated for a subsequent trial^23^. Typically, a between-trial analysis reveals how the variability of motor control changes from trial to trial. These trial-to-trial changes represent higher level cognitive functions (e.g., executive functions) that allow flexible and stable bimanual force control between trials^24,25^.

A critical between-trial analysis examined task stabilization in the motor system by determining specific interlimb coordination strategies across multiple trials^25,26^. This interlimb coordination approach is known as the uncontrolled manifold hypothesis (UCM). The UCM hypothesis posits that the space of elemental variables indicating motor actions (i.e., pairs of left and right mean force for each trial) produced by a redundant system includes two sub-spaces: (a) UCM and (b) orthogonal (ORT) to UCM^23,27^. The UCM is consistent with desired motor solutions so that elemental variables are not “controlled” in this space. Thus, the variance of elemental variables found within the UCM is referred to as good variance (V_UCM_) although this variance does not affect task performances. The amount of V_UCM_ can indicate levels of motor flexibility implying the availability of various motor solutions is effective for achieving task goal in the motor system. However, the variance of elemental variables that are orthogonal to UCM refers to bad variance (V_ORT_) and this variance interferes with task performance. The higher values of V_ORT_ indicate decreased task stability across multiple trials. When executing bimanual force control tasks, the proportion of V_UCM_ relative to V_ORT_ is the index of bilateral motor synergies (V_Index_). Greater values of V_Index_ indicate higher task stability (i.e., improvements in bimanual force control)^28,29^.

According to the UCM hypothesis, the central nervous system (CNS) prefers flexible motor control solutions from various alternatives to stabilize task performance^27,30,31^. For instance, the CNS may control elemental variables (e.g., muscles, joints, and limbs) or multiple effectors in a synergic way rather than selecting and initiating unique motor solutions^23,27,30^. Previous force control studies reported that the presence of visual feedback improved task stability as indicated by UCM variables. Vision influenced the non-motor equivalent components in a four-fingers force control task and V_Index_ in a bimanual index finger force control task as compared with no vision^17,32,33^. Moreover, recent UCM findings indicated that V_Index_ during bimanual force control tasks increased from no vision to vision conditions, and the improved V_Index_ values were related to a reduction of force error and variability within a trial^34^. Beyond these identified vision and no vision effects, potential changes in bilateral motor synergies are still unknown across multiple trials that vary by visual gain.

Importantly, motor synergies and visual gain support the possibility that an altered amount of visual feedback may influence hierarchical multi-levels involved in sending neural control signals that potentially modulate elemental variables stabilizing task performances across multiple trials^27^. Two sets of investigators reported higher cortical activation patterns in the premotor cortex when individuals produced increased motor synergies across bimanual force control trials^35,36^. Interestingly, higher visual gain conditions improved the accuracy of isometric force control with greater cortical activation in the dorsal premotor region and inferior parietal lobule as compared with lower visual gain condition^12,13^. Moreover, prior findings suggested that the activation of cerebellum and basal ganglia may be involved in facilitating flexible force outputs for task stabilization^37,38^. Importantly, previous studies reported that the cerebellum and basal ganglia are related to visual perception as well as fine motor control^39–42^. Specifically, the lateral cerebellum revealed sensitive activation toward intermittent visual gain while participants performed pinch gripping tasks^43^. Further, neuronal populations of the basal ganglia responded to both haptic and visual information manipulations^44,45^. These cumulative findings advanced the hypothesis that a greater amount of visual gain may influence bilateral motor synergies, thus improving force control because of the involvement of key cortical and sub-cortical regions.

The purpose of the current study was to investigate the effects of different visual gain conditions on bilateral motor synergies while participants performed a submaximal bimanual force control task. Four visual gain conditions were manipulated: 8, 80, 256, and 512 pixels/N. These visual gain conditions were randomly presented during the force control task that involved simultaneously abducting the left and right index fingers against load cells. Consistent with previous findings^17,46^, we hypothesized that higher visual information gain would increase bilateral motor synergies. In addition, we predicted that the lowest to highest visual gain conditions would favorably affect force control because of the bilateral motor synergies.

## Results

**Figure 1:**
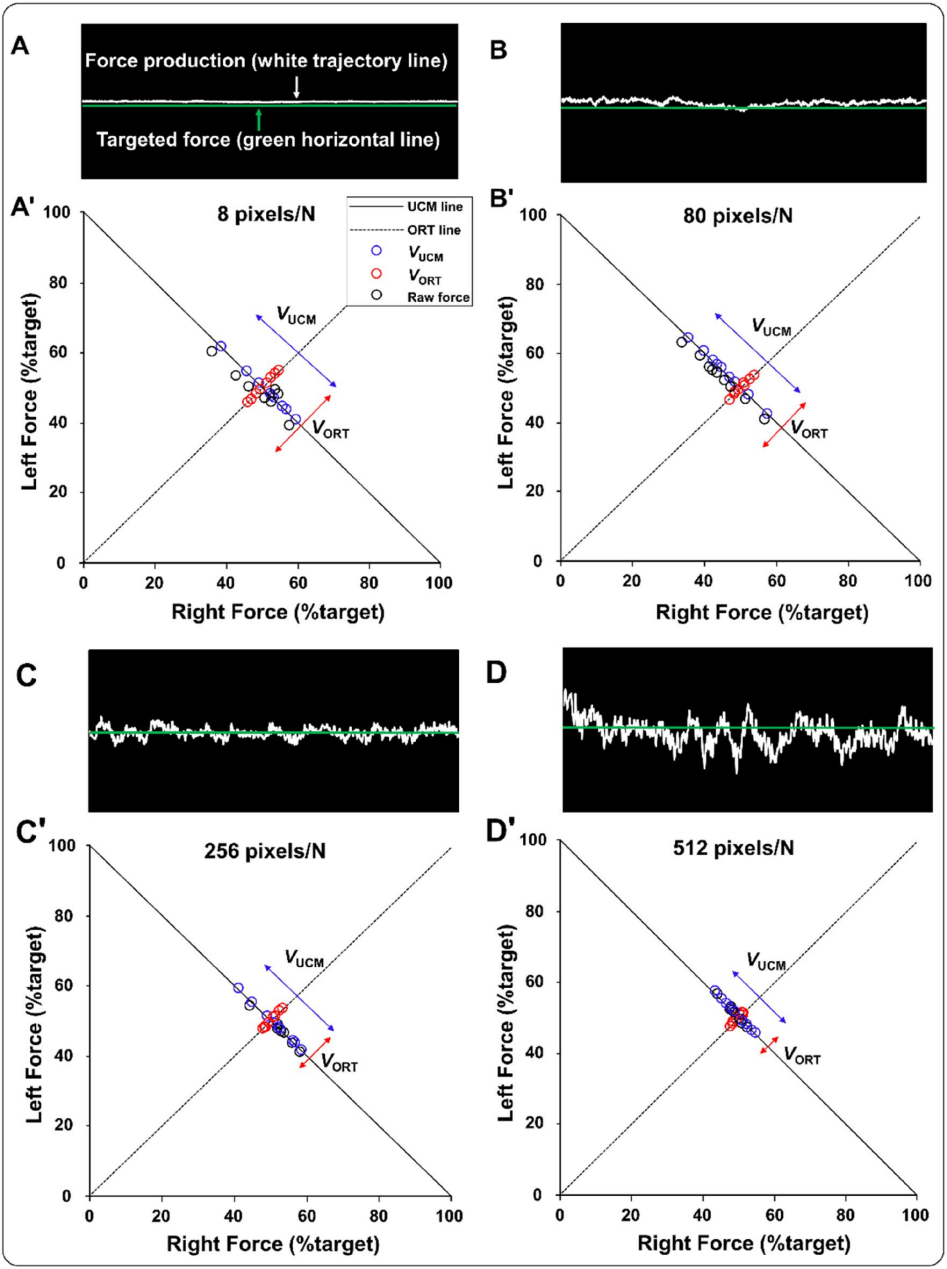
Representative force fluctuation (upper) and UCM data (lower) at visual gain. Force fluctuation figures displays force production (white trajectory line) and targeted force level (green horizontal line). (**A**) 8 pixels/N, (**B**) 80 pixels/N, (**C**) 256 pixels/N, and (**D**) 512 pixels/N. UCM analysis quantifying variances of nine pairs of normalized elemental variables from each trial (i.e., black circles) projected to both UCM (i.e., black line) and ORT (i.e., black dotted line). (**A′**) 8 pixels/N, (**B′**) 80 pixels/N, (**C′**) 256 pixels/N, and (**D′**) 512 pixels/N.

The submaximal force control task involved producing and maintaining total force around the targeted force level. Total force, the summation of left and right forces, was calculated as the submaximal force (i.e., 20% of maximum voluntary contraction: MVC) recorded by the two load cells while both index fingers abducted at the same time. The UCM analysis examined three variables (i.e., V_Index_, V_UCM_, and V_ORT_) and bimanual force control variables included force accuracy, variability, and symmetry. Figure 1 shows representative force fluctuation data for each visual gain condition. Further, representative UCM findings displaying nine pairs of elemental variables (left and right mean forces for each trial) projected to UCM and ORT for each visual gain condition.

### Bilateral motor synergies: UCM findings

**Figure 2:**
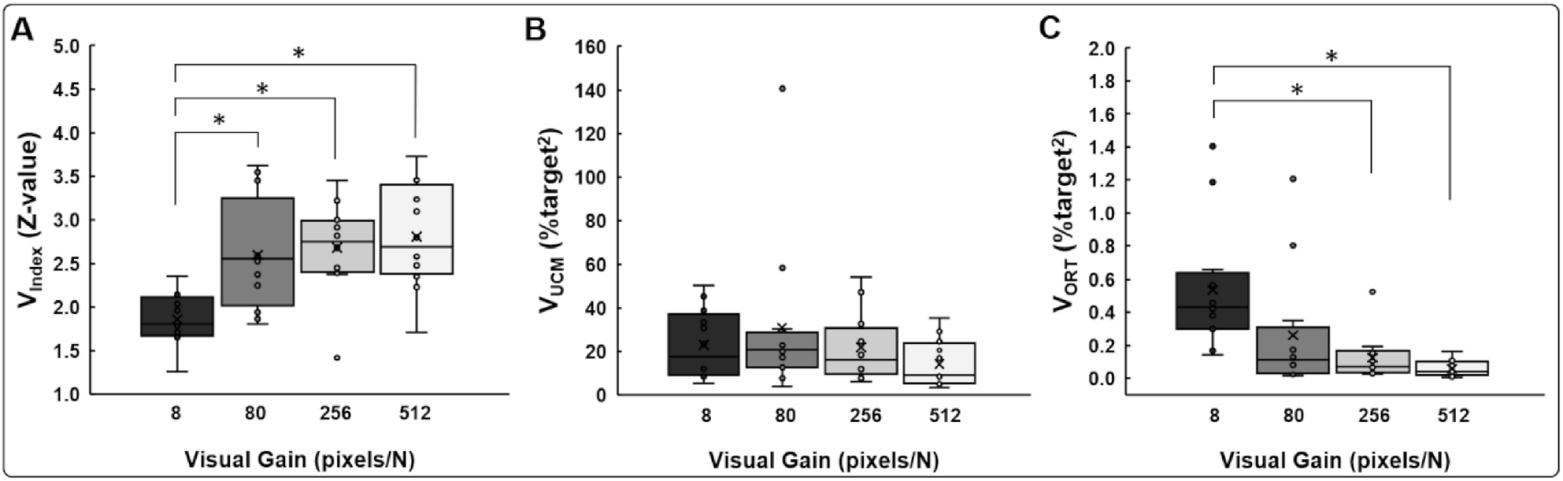
UCM analysis findings. (**A**) Bilateral motor synergies (V_Index_) for different visual gain conditions. (**B**) Good variance (V_UCM_) for different visual gain conditions. (**C**) Bad variance (V_ORT_) for different visual gain conditions. Box plot shows mean (the X inside box), median (the black horizontal line inside box), interquartile range (IQR = Q3 – Q1; top and bottom of box), minimum value: Q1-1.5 × IQR and maximum value: Q1 + 1.5 × IQR (lower and upper error lines), and data falling outside minimum and maximum values (black filled circles). *Asterisk* (*) indicates a significant difference as comparison with 8 pixels/N (*P* < 0.05).

The one-way repeated measures ANOVA on the V_Index_ showed a significant visual gain main effect: [*F*(3, 33) = 10.063; *P* < 0.001; partial η^2^ = 0.478; Fig. 2A]. Post hoc analysis indicated that the values of V_Index_ at 80 pixels/N (*P* = 0.019), 256 pixels/N (*P* = 0.002), and 512 pixels/N (*P* < 0.001) were significantly greater than those at 8 pixels/N. These findings demonstrate that greater bilateral motor synergies were observed in the three highest visual gain conditions (i.e., 80, 256, and 512 pixels/N) than those in the lowest visual gain condition.

The analysis on the V_UCM_ data failed to show a significant visual gain main effect: [*F*(1.313, 14.443) = 2.137; *P* = 0.163; partial η^2^ = 0.163; Fig. 2B]. However, analysis of the V_ORT_ data revealed a significant visual gain main effect: [*F*(2.188, 24.067) = 8.462; *P* = 0.001; partial η^2^ = 0.435; Fig. 2C]. Post hoc analyses revealed that the V_ORT_ at 256 pixels/N (*P* = 0.034) and 512 pixels/N (*P* = 0.006) were significantly decreased in comparison to those at 8 pixels/N. These findings indicated that compared with the lowest visual gain condition the stability of bilateral force control across multiple trials increased in the two highest visual gain conditions (i.e., 256 and 512 pixels/N).

### Bilateral force accuracy, variability, and force symmetry

**Figure 3:**
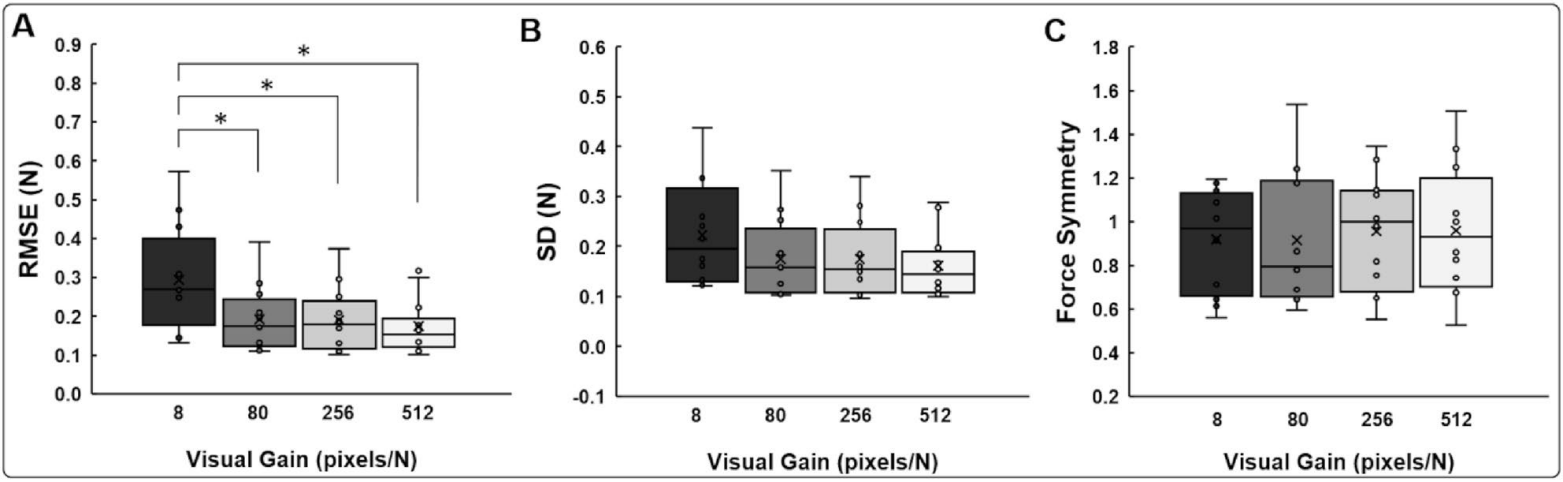
Bimanual Force control findings. (**A**) Force accuracy (RMSE) for different visual gain conditions. (**B**) Force variability (SD) for different visual gain conditions. **(C**) Force symmetry for different visual gain conditions. Box plot shows mean (the X inside box), median (the black horizontal line inside box), interquartile range (IQR = Q3 – Q1; top and bottom of box), minimum value: Q1-1.5 × IQR and maximum value: Q1 + 1.5 × IQR (lower and upper error lines), and data falling outside minimum and maximum values (black filled circles). *Asterisk* (*) indicates a significant difference as compared with 8 pixels/N (*P* < 0.05).

The one-way repeated measures ANOVA on the RMSE revealed a significant visual gain main effect: [*F*(1.270, 13.965) = 12.352; *P* = 0.002; partial η^2^ = 0.529; Fig. 3A]. Post hoc analysis showed that the RMSE at 80 pixels/N (*P* = 0.040), 256 pixels/N (*P* = 0.032), and 512 pixels/N (*P* = 0.009) was significantly lower than at 8 pixels/N. Further, the analysis on the SD showed a significant visual gain main effect: [*F*(1.358, 14.943) = 4.808; *P* = 0.035; partial η^2^ = 0.304; Fig. 3B]. However, the post hoc analyses revealed decreased trends in the SD from 8 to 512 pixels/N (*P* = 0.076). Finally, the analysis on the force symmetry revealed no significant visual gain main effect [*F*(1.450, 15.951) = 0.350; *P* = 0.642; partial η^2^ = 0.031; Fig. 3C]. Additional one sample *t*-tests confirmed that the force symmetry values for each vision gain conditions were not significantly different from 1: (a) 8 pixels/N: *t*_11_ = − 1.242; *P* = 0.240, (b) 80 pixels/N: *t*_11_ = − 1.001; *P* = 0.339, (c) 256 pixels/N: *t*_11_ = − 0.600; *P* = 0.561, and (d) 512 pixels/N: *t*_11_ = − 0.494; *P* = 0.631. Together, these findings demonstrate that greater bimanual force accuracy was observed in the three highest visual gain conditions (i.e., 80, 256, and 512 pixels/N) than those in the lowest visual gain condition and for each visual gain condition, participants produced symmetrical forces between hands.

### Correlations between UCM and bilateral force control variables

**Figure 4:**
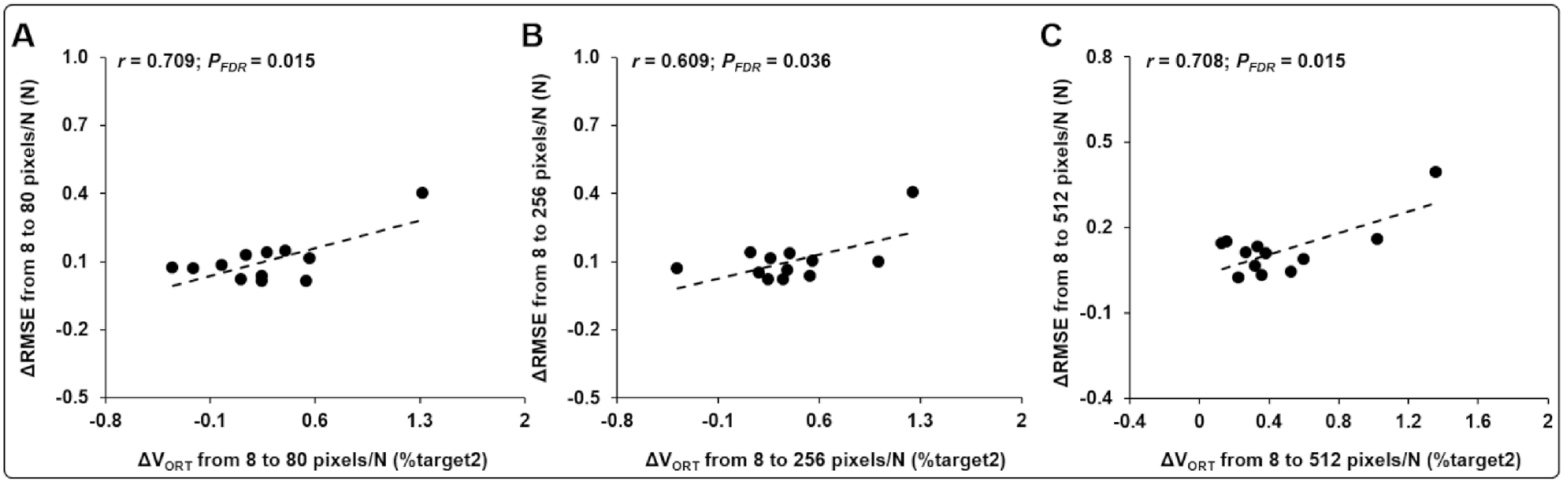
Correlation findings between changes in V_ORT_ and RMSE from the lowest to three higher visual gain conditions. (**A**) Δ in V_ORT_ versus Δ in RMSE from 8 to 80 pixels/N. (**B**) Δ in V_ORT_ versus Δ in RMSE from 8 to 256 pixels/N. (**C**) Δ in V_ORT_ versus Δ in RMSE from 8 to 512 pixels/N. Note that *P*_FDR_ indicates *P* value corrected by a false discovery rate.

The correlation analyses revealed that a reduction of V_ORT_ from 8 to 80 pixels/N were positively related to a decrease in RMSE from 8 to 80 pixels/N. This significant correlation was observed for other two visual gain comparisons (i.e., 8 vs. 256 pixels/N, and 8 vs. 512 pixels/N; Fig. 4; Table 1). For the SD values, we found no significant correlation findings. These results suggest that improvements in stability across multiple trials were significantly associated with an increase in force accuracy within a trial from the lowest to the three highest visual gain conditions, respectively.

**Table 1:** Correlation findings between changes in RMSE and UCM variables.

	8–80 pixels/N ΔV_Index_		8–80 pixels/N ΔV_UCM_		8–80 pixels/N ΔV_ORT_	
	*r*	*P*_*FDR*_	*r*	*P*_*FDR*_	*r*	*P*_*FDR*_
8–80 pixels/N ΔRMSE	–0.148	0.647	0.313	0.647	0.709*	0.015

	8–256 pixels/N ΔV_Index_		8–256 pixels/N ΔV_UCM_		8–256 pixels/N ΔV_ORT_	
	*r*	*P*_*FDR*_	*r*	*P*_*FDR*_	*r*	*P*_*FDR*_
8–256 pixels/N ΔRMSE	–0.153	0.647	0.148	0.647	0.609*	0.036

	8–512 pixels/N ΔV_Index_		8–512 pixels/N ΔV_UCM_		8–512 pixels/N ΔV_ORT_	
	*r*	*P*_*FDR*_	*r*	*P*_*FDR*_	*r*	*P*_*FDR*_
8–512 pixels/N ΔRMSE	–0.331	0.647	0.183	0.647	0.708*	0.015

*FDR* false discovery rate.

*P_*FDR*_ < 0.05.

## Discussion

This study investigated the effects of different levels of visual gain (i.e., 8, 80, 256, and 512 pixels/N) on bilateral motor synergies and bimanual force control. In comparison to the lowest visual gain condition, greater bilateral motor synergies and bimanual force accuracy were identified in the three highest visual gain conditions. Better stability of bilateral force control across multiple trials as indicated by lower bad variance appeared in the two highest visual gain conditions (i.e., 256 and 512 pixels/N) as compared with the lowest visual gain condition. Further, the correlation findings indicated that the reduction of bad variance from the lowest to the three highest visual gain conditions was related to better bimanual force accuracy.

Consistent with our hypothesis, the UCM analysis revealed more motor synergies between the two hands in the three highest visual gain conditions (i.e., 80, 256, and 512 pixels/N) than those in the lowest visual gain condition (i.e., 8 pixels/N). Beyond the prior findings that reported better interlimb force coordination patterns with an increased amount of visual feedback within a trial^12^, our results indicated that different levels of visual gain changed bimanual coordination patterns across multiple trials. Although the UCM approach indirectly assess motor synergies by quantifying a proportion of V_UCM_ relative to V_ORT_, combined our V_Index,_ V_UCM_, and V_ORT_ findings indicate that how the motor system stabilizes performance variables across multiple trials^23,29^. To stabilize task performances (i.e., greater V_Index_), the motor system may select one of three strategies: (a) increased flexibility (higher V_UCM_) of elemental variables without changes in stability (V_ORT_), (b) increased stability (lower V_ORT_) of elemental variables without changes in flexibility (V_UCM_), and (c) increased both flexibility (higher V_UCM_) of elemental variables and stability (lower V_UCM_). Our recent findings revealed that the presence of visual feedback using two visual gain conditions (i.e., 13 pixels/N at 5% of MVC and 8 pixels/N at 25% and 50% of MVC) improved bilateral motor synergies with decreased bad variance as compared with no vision condition^34^. These findings suggested that the availability of visual information during bimanual force control may facilitate the motor system to increase the stability patterns (i.e., a reduction of bad variance) across multiple trials stabilizing task performances. Similarly, despite no significant changes in V_UCM_, we found a distinct pattern showing that higher visual gain conditions contribute to a reduction of V_ORT_ that may stabilize bilateral force control. These findings imply that the increased visual gain may increase stability (i.e., decreased V_ORT_) across multiple trials and subsequently for improving bilateral motor synergies. This explanation is consistent with the conclusion that the environmental visual information strongly influenced interlimb force coordination patterns^9^.

Not surprisingly, the three highest visual gain conditions (i.e., 80, 256, and 512 pixels/N) improved bimanual force control with reduced force error compared with those in the lowest visual gain condition (i.e., 8 pixels/N). This evidence indicates that the higher amount of visual feedback successfully contributed to task stability within a trial, and this is consistent with previous findings^14,15^. Moreover, our correlation findings indicated that a decrease in V_ORT_ across multiple trials with higher visual gain conditions was significantly related to less force error within a trial. These findings expand prior results in that bilateral motor synergies across multiple trials from no vision to vision conditions were associated with improved bimanual force control within a trial^34^. Improved bilateral force control strategies with increased visual gain is typically observed when a surgeon is performing an operation using microscopic surgery techniques^47,48^. Although no extreme value was identified in the correlation findings based on the outlier exclusion method (mean ± 3 × SD)^49–53^, these correlation patterns should be cautiously interpreted because of the potential effects of the variables (i.e., visually suspected outlier in Fig. 4). Thus, we recommend that additional studies be conducted with increased sample size tested for consolidating the relationship between changes in bad variability and force accuracy from lowest to highest visual gain condition.

Importantly, our findings revealed that bilateral motor synergies and bimanual force control improved for the three highest visual gain conditions in comparisons to the 8 pixels/N, and these motor control improvements plateaued beyond 80 pixels/N. When the amount of visual information is relatively low, individuals may predominately rely on proprioceptive feedback during movement execution^54,55^. The limited external feedback resources such as visual information across multiple trials presumably impaired bilateral motor control and motor synergies (e.g., reduced V_ORT_ without altered V_UCM_)^56,57^. Previous studies reported that a greater amount of visual information beyond a certain level (e.g., > 1°) did not dramatically affect force control^13,58^. Similarly, healthy young adults showed no significant changes in force variability from 64 to 1424 pixels/N^15^. Moreover, an fMRI study reported neural activity in key cortical regions related to visuomotor processing including the primary motor cortex and visual cortex. Activation increased with a reduced force error while processing increased level of visual feedback below 1° of visual angle^13^. Beyond 1° of visual angle, participants showed no significant changes in force error or cortical activations in the visuomotor areas. These findings provided a possibility that performers may be more dependent on proprioceptive information at the lowest visual gain conditions. Alternatively, when visual information is increased to a sufficient level, performers apparently prefer online visuomotor motor corrections for stabilizing task performances^15^. Presumably, these findings suggest that positive effects of increased visual gain on bilateral motor synergies and bimanual force control may be limited to a certain level (e.g., 80 pixels/N).

When visual gain levels increased, greater bilateral motor synergies in the motor system may be associated with the premotor areas as well as cerebellum and basal ganglia^29,59,60^. Increased cerebellar activations may be involved in motor error correction and flexibility of motor synergy formation^25,37^. Moreover, the neural pathway from the inferior olive to cerebral cortex was highly facilitated when lower movement errors appeared across multiple trials of center-out reaching tasks performed by primates^61,62^. The presence of visual feedback improved isometric force control via facilitated visuomotor processing in the lateral cerebellar regions where the dentate nucleus may regulate fine motor control using external stimuli^63^. Further, when higher visual gain was provided, greater cortical activation appeared in the premotor regions including ventral and dorsal premotor areas connecting to the cerebellum contributing to successful force control^64,65^. Moreover, the basal ganglia contributes to fine motor control by processing the information on upcoming movement received from the cerebral cortex^66^. The visual information improves phasic responses of dopaminergic neurons in the substantia nigra and ventral tegmental area of the basal ganglia system resulting in greater dopamine concentrations in the striatum and nucleus accumbens during visual perception^67,68^. Potentially, the positive effects of higher visual gain conditions on bilateral motor synergies across multiple trials may be associated with improved online-motor corrections via communication between key cortical and sub-cortical regions^13^.

Despite the positive effects of higher visual gain conditions on bilateral motor synergies and bimanual force control, the current findings should be cautiously interpreted. First, although a recent study recommended approximate four trials as a minimum number of trials for quantifying V_Index_, reliable estimations on the variance of elemental variables within the sub-spaces (e.g., V_UCM_) require 10–14 trials while performing multi-finger motor actions controlling force control across trials^69^. Given that participants in the current study performed nine bimanual force control trials for each visual gain condition, we recommend that the V_ORT_ findings need to be further investigated in future studies with a sufficient number of trials. Second, given that visual gain effects on isometric force control were observed in unimanual and bimanual conditions^9,14^, future studies need to determine whether increased visual gain improved UCM variables within a unimanual hand (e.g., multi-finger force production). In addition, Lee Hong and Newell suggested that the temporal structure of force variability could be influenced by the interaction of visual gain and targeted force levels^70^. Presumably, an optimal level of visual gain may be dependent on targeted force levels causing altered force variability. Although we found that the three highest visual gain conditions revealed greater bilateral motor synergies than those in the lowest visual gain, these patterns were limited to bimanual force control at 20% MVC. At the 50% MVC that may induce more variable force outputs, the lowest visual gain (e.g., 8 pixel/N) may be optimal for improving bilateral motor synergies. Thus, determining optimal visual gain levels depending on a range of targeted force levels would further advance the UCM hypothesis. Finally, based on several UCM findings that raised the possibility of involving key cortical and sub-cortical brain areas in greater bilateral motor synergies^59,60,71^, further brain imaging studies are necessary to confirm potential neurophysiological mechanisms underlying altered bilateral motor synergies by quantifying changes in functional activities of the cortical and sub-cortical areas across multiple visuomotor control tasks.

## Conclusion

In conclusion, the current study revealed that the three highest visual gain conditions improved bilateral motor synergies and bimanual force control than the lowest visual gain condition. Further, consistent with the UCM hypothesis, decreased bad variance was related to increased force accuracy from the lowest to the three highest visual gain conditions. These findings indicate that the increased levels of visual gain may effectively facilitate advanced motor synergic patterns between two hands across trials and task stabilization. Given that the optimal bimanual movement control may be influenced by three constraints pertaining to the organism, environment, and task constraints^9,72^, further studies should investigate the interactive effects of different participants (e.g., individuals with neurological diseases) and levels of task difficulty (e.g., asymmetry force control) on bilateral motor synergies and task stabilization patterns.

## Methods

### Participants

Twelve healthy young individuals (mean age ± standard deviation = 25.6 ± 5.6 years; 12 right-handed) participated in this study. No participants had any musculoskeletal impairments in their upper extremities, neurological deficits, and non-corrected impairments of vision. We determined the sample size by conducting a priori power analysis based on the pilot data using G*Power software (version 3.1.9.7)^73^. The analysis confirmed that 12 participants in a within-subjects design were minimally required (power = 0.99 and alpha = 0.05). The current study protocols were approved by the University of Florida’s Institutional Review Board, and we confirmed that all methods were performed in accordance with the relevant guidelines and regulations. Before starting the test, all individuals read and signed an informed consent.

### Experimental procedures

To perform bimanual isometric force control tasks, participants sat in an upright position on a chair facing 78 cm away from a 43.2 cm LCD monitor (100 Hz refresh rate). The monitor had 768 pixels vertically, and 1024 pixels horizontally. We instructed participants to comfortably place both of their arms in a prone position on the table (elbow flexion = 20°–40° and shoulder flexion = 15°–20°). Task performance involved participants executing bimanual index fingers’ abduction against two separate force transducers (MLP-25; Transducer Techniques; 4.16 × 1.27 × 1.90 cm, range = 11 kg, 0.1% sensitivity). Participants’ wrist and other fingers were secured in a locked position to prevent unintentional force productions.

Initially, participants performed three MVC trials to determine each individual’s targeted force level. For each MVC trial, participants generated a total forces (i.e., the sum of forces simultaneously produced by both index fingers) as much as possible for 6 s. Further, the concurrent visual information on their total forces (i.e., the white trajectory line) was provided during MVC tasks. For each MVC trial, we identified a value of peak force and then calculated mean of the three peak forces as MVC level for each participant. We set 20% of MVC as a targeted force level for the submaximal bimanual force control tasks. The goal of the submaximal force control task was to continuously match the sum of bimanual isometric forces generated by the left and right index fingers (i.e., the white trajectory line) to a targeted force level (i.e., green horizontal target line) for 15 s. During all MVC and submaximal force control trials, we instructed participants to produce bimanual forces from both hands, and confirmed compliance on an oscilloscope.

Although previous studies reported that isometric force control improved from 8 to 80 pixels/N^9,15^, there is evidence missing on whether visual gain above 80 pixels/N influences bimanual force control and bilateral motor synergies. The unimanual force control study reported no significant reduction of force variability from moderate-to-high visual gains (e.g., 64–1424 pixels/N)^15^, whereas interlimb force coordination, as indicated by correlation coefficient between left and right forces was improved from 80 to 256 pixels/N^10^. Moreover, UCM studies focused changes in bilateral motor control strategies across multiple trials under a fixed visual gain condition (< 50 pixels/N)^17,74^. Thus, we manipulated the amount of visual information by providing four different levels of visual gain (i.e., 8, 80, 256, and 512 pixels/N) during the submaximal force control task. In particular, we included 512 pixels/N as the highest visual gain because this level was maximal range of visual information in the screen (768 vertical pixels/512 pixels/N = 1.5 N) that can display upper and lower limits of force variability produced by both index fingers at 20% of MVC (e.g., SD of force < 1 N) reported in prior studies^9,10^. As the level of visual gain increased, participants received greater amounts of visual information within a trial.

A recent study that examined a minimal number of trials specifically for multi-finger isometric force data recommended at least four trials for quantifying the synergies and V_Index_^69^. Given that the primary interest of this study focused on changes in bilateral motor synergies across visual gain conditions, we administered nine submaximal force control trials for each of the four visual gain blocks. The visual gain blocks were presented randomly, and all participants executed 36 total submaximal force control trials. To minimize muscle fatigue, we provided two rest intervals: (a) 10 s between trials and (b) 60 s between visual gain blocks. A custom LabVIEW program (National Instruments, Austin, USA) administered the experimental procedures and collected the data.

### Data analyses

A 15TL Grass Technologies Physio-data Amplifier System (AstroMed Inc., an excitation voltage of 10 V and a gain of 200) amplified and sampled force signals at the sampling rate of 100 Hz using a 16-bit analog-to-digital converter (A/D; NI cDAQ–9172 + NI9215, force unit detected minimally 0.0016 N). Using a bidirectional fourth-order Butterworth filter with a cut-off frequency of 20 Hz, force data were filtered. Allowing participants to make force production adjustments of 3 s at movement initiation and 1 s at movement termination provided a clean set of data for the middle 11 s of force control. This minimized potential force production errors at the beginning and end of movements. All offline data analysis procedures were performed using a custom Matlab program (Math Works™ Inc., Natick, USA).

Consistent with previous UCM studies^27,30^, we quantified bilateral motor synergies based on the UCM hypothesis. First, we averaged values of force outputs produced by left and right index fingers for each trial, respectively. Second, we normalized two mean values relative to the targeted force level and set a pair including two normalized unimanual index finger forces as an elemental variable for each trial. Third, we performed the same procedures across the nine trials given that nine total elemental variables were acquire in the visual gain condition (block). Then, the elemental variables were projected to UCM and ORT sub-spaces, respectively (Fig. 1).

The variances of elemental variables within UCM is V_UCM_ and the variance of elemental variables within ORT is V_ORT_. Total variance (V_TOT_) is a sum of V_UCM_ and V_ORT_. Based on Eq. (1), for each visual gain block, we quantified the index of bilateral motor synergies, and additionally we performed a Z-transformation for further parametric analysis (Eq. 2). Thus, higher values of V_Index_ indicated higher task stability across multiple trials^30,75^.

1 $${V}_{Index}=\frac{{V}_{UCM}/{df}_{UCM}-{V}_{ORT}/{df}_{ORT}}{{V}_{TOT}/{df}_{TOT}}$$

2 $${V}_{Index}\left(Z-transformed\right)=0.5\times ln\frac{2+{V}_{Index}}{2-{V}_{Index}}$$

where the degrees of freedom for good variability (df_UCM_) and bad variability (df_ORT_) is 1 and the degrees of freedom for total variability (df_TOT_) is 2. Consistent with the UCM hypothesis the values of V_Index_ range from − 2 to 2.

To estimate bimanual force control within a trial, we analyzed force accuracy, variability, and force symmetry. Consistent with traditional analyses, we calculated root-mean squared error (RMSE) and standard deviation (SD). These calculations provided within a trial force accuracy and variability values. In addition, we estimated force symmetry by calculating the ratio of left mean force relative to right mean force for each trial. The values close to 1 indicates symmetrical force production between hands, whereas the values less than 1 denotes greater force production by right hand than those by left hand.

To determine the effects of altered visual gain on bilateral motor synergies, force control, and force symmetry, we conducted one-way repeated measures ANOVAs on the six dependent variables (V_Index_, V_UCM_, V_ORT_, RMSE, SD, and force symmetry). The four levels of visual gain conditions 8, 80, 256, and 512 pixels/N were analyzed in a within-subjects design. Normality of the force data was confirmed using the Shapiro–Wilk’s W test^76^. When assumptions of sphericity were violated, we report Greenhouse–Geisser’s conservative degrees of freedom adjustment. For post hoc analysis, we used Bonferroni’s pairwise comparisons. For the force symmetry values of each visual gain condition, we additionally conducted one sample *t*-test to specifically determine whether bilateral forces were symmetry between hands.

Moreover, we performed the correlation analyses to determine whether changes in UCM variables (i.e., V_Index_, V_UCM_, and V_ORT_) in three higher visual gain conditions (i.e., 80, 256, and 512 pixels/N) as compared with those in the lowest visual gain condition (i.e., 8 pixels/N) were related to changes in bimanual force control task stabilization (i.e., RMSE and SD). For example, we quantified altered values in V_Index_ and RMSE (i.e., mean of RMSE values from nine bimanual force control trials for each visual gain condition) from 8 to 80 pixels/N for each participant. After conducting the same calculations for all participants, we measured the strength of the linear relationship between two variables (i.e., 12 values of Δ in V_Index_ and 12 values of Δ in RMSE) using Pearson’s correlation analysis. Similarly, we measured the correlations for (a) 12 values of Δ in V_Index_ and 12 values of Δ in RMSE from 8 to 256 pixels/N and (b) 12 values of Δ in V_Index_ and 12 values of Δ in RMSE from 8 to 512 pixels/N. Taken together, these correlations were conducted for the following six comparisons, respectively: (a) Δ in V_Index_ versus Δ in RMSE, (b) Δ in V_UCM_ versus Δ in RMSE, (c) Δ in V_ORT_ versus Δ in RMSE, (d) Δ in V_Index_ versus Δ in SD, (e) Δ in V_UCM_ versus Δ in SD, and (f) Δ in V_ORT_ versus Δ in SD.

All statistical tests were conducted with alpha set at the conventional level (α = 0.05). For post hoc analyses, we conducted Bonferroni’s test adjusted for the number of multiple follow-up comparisons. The statistical analyses were performed using IBM Statistics 22 (SPSS Inc., Chicago, LL; www.ibm.com). Finally, to correct multiple comparisons in the correlation findings we used a false discovery rate (FDR) of *P* < 0.05^77^.

## Data Availability

All data generated or analysed during this study are included in this published article.
